# Oblivious?

**Published:** 1871-08

**Authors:** 


### OBLIVIOUS?

In the April number of the *Dental Cosmos*, Dr. A. C. Castle remarks, "The city of Baltimore and the city of Philadelphia, have the honor of having inaugurated dental schools."

Probably the doctor never heard of Cincinnati—a village lying at the northern end of the longest wire bridge in the world. And may be he didn't know that the said village had a dental school before the said city of Philadelphia. And possibly he didn't know that the said school gave the first course of lectures on dental chemistry, the Christian philosopher, Elijah Slack, being the lecturer. And doesn't know that the two leading text-books were written by the alumni and professors of the said school. How could he? He was not writing "notes for a memoir" of dental colleges. W.

